# PD240 Report Of Outcomes In Health Technology Assessment For Technologies In Ultrarare Diseases In The Brazilian Public Health System

**DOI:** 10.1017/S0266462324004574

**Published:** 2025-01-07

**Authors:** Denis Satoshi Komoda, Juliana Machado-Rugolo, Daniel da Silva Pereira Curado, Silvana Molina Lima, Luis Gustavo Modelli de Andrade, Lehana Thabane, Silke Anna Theresa Weber, Marisa Santos, Monica Aparecida de Paula de Sordi, Marilia Mastrocolla de Almeida Cardoso

## Abstract

**Introduction:**

Ultrarare diseases (URD) represent a challenge to health technology assessment (HTA). The traditional framework for assessing efficacy and cost effectiveness may be biased to include clinically relevant outcomes, leaving patient-centered outcomes doomed to neglect. Here we explore patient-centered outcomes in the context of patient and citizen involvement in the assessment of URD by the Brazilian National Committee for Health Technology Incorporation (CONITEC).

**Methods:**

We assessed 53 HTA reports from CONITEC that evaluated URD-related technologies (and included highlights of patients’ and citizens’ perspectives during recommendation meetings) published from 2012 to 2022. Data extraction was performed by two independent researchers. Data on year of report, sex, ethnicity, category (patient or family), and previous experience with the assessed technology were extracted and analyzed using descriptive statistics. Patients’ and citizens’ narratives were collated from the reports. A thematic analysis was conducted according to patient-centered outcomes and technology-related outcomes and was then compared with the evidence synthesis protocol described in the HTA.

**Results:**

Only seven URD-related HTA reports registered patient or citizen participation, all of which were published in 2022. The age of two participants was reported (both 17 years). Six participants were women. Ethnicity was not reported. All participants had previous experience with the technology. Four participants were family or caregivers and three were patients. Considering patient-centered outcomes, physical (muscular strength) and emotional (self-confidence) improvements that positively affected independence in basic daily functions were reported. These functions included activities such as dressing, self-care, cooking, and leisure. Advantages listed for the assessed technologies included the possibility of self-administration of medication (e.g., swallowing a pill, opening a medicine bottle, and using a syringe).

**Conclusions:**

The results show that although, in some cases, primary outcomes reported in evidence synthesis protocols include patient-centered outcomes (e.g., activities of daily living), in other cases the evidence synthesis failed to identify relevant studies. In other cases, the reports failed to differentiate between primary and secondary outcomes or to fully account for patient-centered outcomes.

